# Influence of Increased Freedom of Movement on Welfare and Egg Laying Pattern of Hens Kept in Aviaries

**DOI:** 10.3390/ani12182307

**Published:** 2022-09-06

**Authors:** Eleonora Nannoni, Giovanni Buonaiuto, Giovanna Martelli, Gabriele Lizzi, Giacomo Trevisani, Gloria Garavini, Luca Sardi

**Affiliations:** 1 Department of Veterinary Medical Sciences (DIMEVET), Via Tolara di Sopra 50, 40064 Ozzano Emilia, Italy; 2 Gruppo Eurovo, Via Ugo La Malfa 15, 40026 Imola, Italy

**Keywords:** laying hens, welfare, behaviour, aviary systems, egg quality, nest use

## Abstract

**Simple Summary:**

The laying hen industry is shifting from cages to alternative housing systems (such as aviaries) to address societal concerns regarding animal welfare. However, it is important to understand the hens’ needs in these systems, in terms of space use and behaviour, to promote their welfare and avoid drawbacks. Therefore, we examined the effects of three different structural modifications (addition of ramps and/or removal of internal partitions) to increase the hens’ freedom of movement in a commercial aviary system. Higher freedom of movement resulted in positive effects on feather conditions and the possibility of choosing between the aviary tiers according to the hens’ preferences. Laying hen producers may therefore adopt some of these structural modifications in aviary systems to enhance the welfare of hens.

**Abstract:**

This work investigates the effects of structural modifications on the welfare level and laying patterns of hens in a three-tier commercial aviary system. Four experimental groups were used: C (control, housed in a traditional aviary); LM (longitudinal movement, in which internal partitions were removed); VM (vertical movement, in which ramps were installed); and FM (freedom of movement, both LM and VM modifications). Hens showed worse body condition scores (*p* < 0.05) in all the modified aviaries, while plumage condition was improved in FM but worsened in VM (*p* < 0.05). No significant effect was observed on egg deposition patterns, egg quality or keel bone damage. When ramps were available (VM and FM groups), hens reduced the number of flights and increased the number of walks from 0.52 to 7.7% of the displacements on average (*p* < 0.05). Apart from some feather pecking concerns in VM (likely due to overcrowding in some favourite aviary areas), LM and FM seemed to facilitate animal movement and promote species–specific behaviour. It is concluded that hen welfare in aviary systems can be improved by means of tailored structural modifications. Producers may therefore adopt some of these modifications (providing ramps and/or removing vertical barriers) to enhance the welfare of hens.

## 1. Introduction

As a result of societal concern over hens’ welfare [1] and European Union legislation banning conventional cages [2], many farmers adopted alternative housing systems [3,4]. At present, more than 190 million laying hens are housed in non-cage systems [5], and the aviary (also called multi-tiered) system is one of the most used, as an alternative to enriched cage systems [6]. Aviaries consist of a littered ground floor and a metal structure with up to four tiers, with nest boxes, drinkers, and perches located on one or more tiers [7,8]. The aim is to make the third dimension accessible to the hens and to divide the environment into different functional areas while also increasing the stocking density compared to single-level systems [8]. This environmental complexity provides the hens with more space and opportunities to perform their natural species-specific behaviour (such as walking/running, dustbathing, wing flapping, perching and flying) compared to enriched cage environments [9,10]. However, aviary design and management vary considerably, which affects how animals distribute in space and their nesting behaviour [11].

Although freedom of movement increases the potential for good welfare of laying hens in multi-tiered systems, some welfare concerns have emerged over the years. These problems are mainly related to an increased prevalence of keel bone distortions/fractures [12,13,14,15], foot pad disorders—hyperkeratosis, dermatitis and bumble foot—[16], and feather pecking/cannibalism, especially when animals are kept at high stocking densities and with intact beaks [17,18]. Keel bone fractures are often caused by collisions of animals against structures or failed landings [15], provoke chronic pain and are detrimental to egg production and nest use, thereby negatively affecting egg quality and labour requirements [19]. Foot disorders may be caused by erroneous perch design and by wet litter [20], and the installation of ramps between aviary tiers has been found to reduce both foot lesions and keel bone fractures in experimental settings [20] and lead to voluntary use of ramps and rapid use of the upper tiers of the aviary [21]. Concerning feather pecking, several studies agree on the positive effect of the provision of litter and/or environmental enrichment materials (as reviewed by Schreiter et al. [22]). Still, minimal information is available on the effects of ramps and the use of space within an aviary on feather pecking [23].

Several commercially available aviaries with fully overlapping tiers have transversal partitions that limit the possibility for animals to move longitudinally across the entire length of the aviary while remaining in the space between the tiers. Animals can overcome these partitions only by moving around them, either by passing on the perches, flying or using the ground floor. While these partitions may have a positive role in limiting overcrowding/piling and promoting division in colonies within large groups (therefore facilitating also group stability and a more uniform egg distribution), they may constitute local obstacles and limit animals’ capability to move to other areas when competitions arise [24].

In recent years, animal welfare associations promoted a Europe-wide citizens’ initiative, End the Cage Age, to stimulate the progressive phasing out of cages across animal species. In laying hens, a further suggestion was to trial different structural changes in commercially available aviaries, such as removing structural elements that restrict or confine (even if temporarily) the birds (i.e., transversal partitions within the tiers, and use of temporary doors in combi systems), and installing ramps to facilitate the movement between the tiers [25]. As highlighted above, while on the one hand increasing the freedom of movement can be considered, by itself, a positive welfare attribute, on the other hand, the complete absence of obstacles within an aviary may increase the risk of overcrowding in some areas (with consequences on nest use, mislaid eggs, and feather pecking) and determine an increased risk of falls/collisions and keel bone damage. Therefore, the trade-offs between these two instances (freedom of movement and absence of lesions) needs to be carefully studied before proposing structural changes to hen farmers. Unfortunately, there is a lack of studies in this area, and particularly on bird movement within commercial aviaries [26]. To this aim, the present work tested the welfare consequences of three different structural modifications to increase hens’ freedom of movement.

## 2. Materials and Methods

The trial was carried out on a commercial farm located in Northern Italy from July 2020 until October 2021. Animals were raised in full compliance with the EU legislation, i.e., Council Directive 1999/74/EC [2]. The trial was authorized by the Ethical Committee of the University of Bologna (Authorization Protocol n° 43819).

Animals were raised on the commercial farm, and periodic farm visits were scheduled approximately every 16 weeks to collect data on egg production and nest use, animal welfare and egg quality. Additionally, some video recordings were made to assess animal behaviour.

### 2.1. Animals and Housing Conditions

Approximately 12,000 laying hens (Lohmann LSL—Classic), originating from the same pullet unit and parent flock, were housed in eight aviaries within the same commercial farm. Since the farm building consists of two floors (the first and the second floor), the experimental aviaries were equally distributed across the two floors of the building.

In the pullet farm, hens were raised in a system similar to the one used in the laying farm (multi-tier aviary, as described below). No ramps were provided to the pullets in this phase, and animals were kept at a stocking density of 29 pullets/m^2^.

In the laying farm, hens were housed in combination aviaries, also called “Combi systems”. This aviary system develops on three tiers (plus the ground floor). Feed, water, nests, perches, egg and droppings collection belts are available on each tier. On the ground floor, concrete blocks were provided as pecking/enrichment material and replaced when consumed. Chopped straw was also provided on this floor.

Stocking density was 22 hens/m^2^ of ground available (i.e., 1495 hens were housed in each aviary). The combi system in use at the farm consisted of aviaries that were 18 m long each. Despite being a unique space across which hens could move freely, these aviaries had internal partitions (four per tier) that divided each tier into five compartments (each compartment measured 3.6×1.1 m). Each compartment contained one group nest, eight drinkers and the feeding line. Each replication (i.e., aviary) consisted of two sides (right and left) and three tiers (high, medium and low), for a total of 30 compartments (15 compartments/side), plus the ground floor covered in litter. Figure S1 (provided in the Supplementary Materials) schematically shows the main features of the aviary.

Temperature and humidity were centrally and automatically kept constant by computerized ventilation and cooling system. During the summer months (July), the recorded temperature inside the building was 22.5 °C, with 56.7% RH. Ventilation had a longitudinal direction.

The lighting and feeding programmes were those usually adopted by the farm, and they were the same for all the animals in the building. Hens were vaccinated with the standard protocol of the pullet farm.

### 2.2. Experimental Design

The trial included two replications (i.e., aviaries) for each of the four experimental groups. To avoid possible confounding effects due to any slightly different conditions on the two floors of the building, each group included one aviary located on the first floor and one on the second floor of the building. For the same reason, the location of the experimental aviary within each floor was chosen to balance spatial distribution and position with respect to the ventilation units. The first and the last aviaries of the row were also avoided.

The 4 experimental groups were:

- Control group (C): animals were kept in standard combi aviaries with no structural modifications. These aviaries have some partitions within the tiers (four on each tier) that are designed to limit the horizontal movement of the animals, thereby reducing the risk of overcrowding (Figure S2);
- Longitudinal Movement group (LM): the aviary was modified by removing all the internal partitions to guarantee an increased possibility of movement along the aviary (longitudinal movement) (Figure S3);
- Vertical Movement group (VM): the aviaries were modified by adding ramps between the tiers to facilitate the hens’ vertical movement across different tiers. Four bottom-to-top ramps were added to each aviary (two on the left and two on the right side). Each ramp was made of three horizontal portions (positioned on top of the perches) linked using two sloped grilled metal surfaces connecting consecutive tiers (Figures S4 and S5);
- Freedom of Movement group (FM): both the modifications presented in LM and VM groups were applied to facilitate both longitudinal and vertical movement (LM + VM).

Pictures showing the modifications made in the aviaries are provided in the Supplemental Materials File (Figures S2–S5).

### 2.3. Data Collection

Farm visits for data collection were scheduled approximately every 16 weeks (at 32, 49, 65 and 78 weeks of age of the hens). On each of these occasions, a group of six trained operators (max three people at the time per aviary) entered the aviaries and collected data on animal welfare and egg distribution. A sample of eggs was also collected for subsequent egg quality assessment. Video recordings for behavioural observations were carried out in the same weeks (a few days before the farm visit).

At the beginning of the trial (16 weeks of age), hens were monitored for welfare (immediately after transfer from the pullet farm to the laying house).

Lastly, farm operators periodically recorded animal weight (weekly weighing of 25 hens per aviary until 35 weeks of age, then monthly weighing) and took note of animals found dead according to the practices in place at the farm.

#### 2.3.1. Welfare Indicators

The same trained group of six people assessed animal welfare and body condition for the entire trial. At the beginning of each farm visit, the assessors evaluated together a group of 10 hens to ensure consistency between evaluators.

Seventy animals were randomly sampled in each experimental aviary (N = 560 for each farm visit). To avoid possible confounding effects due to the hierarchy of the animals and sampling technique, the sample was stratified by picking up, in each aviary, 10 animals from the ground and 20 from each of the three tiers. Animals were picked up in equal numbers from both the left and right sides, and along the entire length of the aviary. The sampling started from the ground and moved to progressively higher tiers to avoid the risk of catching the same animal twice.

On each of the sampled hens, the following parameters were assessed:

- Body Condition Score (BCS) was assessed as an indicator of good feeding: keel bone prominence was estimated both visually and by running fingers alongside and over the keel bone. BCS was scored as 0 = emaciated (severely prominent keel, depressed contour to breast muscle); 1 = lean (slightly to moderately prominent keel, but does not feel sharp); 2 = normal (smooth to moderate breast muscle contour with keel);
- Keel Bone Damage (KBD) was assessed as an indicator of pain and lesions due to collisions with the aviary structures, according to the Welfare Quality assessment protocol for laying hens [27]: 0 = no deviations, deformations or thickened sections, keel bone completely straight; 1 = deviations (flattening, s-shape, bending) or thickened sections present in slight form; 2 = evident deviation or deformation of keel bone (including thickened sections);
- Plumage damage was assessed on 6 regions of the body (neck, breast, back, belly, tail and wing), and each region was scored as follows: 0 = intact plumage; 1 = slight wear, nearly complete feathering; 2 = moderate wear, a few broken feathers (tail, wing) or damaged feathers leaving featherless areas less than 5 cm in diameter (in the other regions); 3 = several broken or missing feathers (tail, wing) or featherless area ≥ 5 cm in diameter (in the other regions); 4 = featherless area ≥ 5 cm in diameter and/or presence of skin lesions;
- Foot pad lesions (bumblefoot) and toe damage: for foot pad lesions 0 = feet intact, no wounds; 1 = no or moderate swelling, not dorsally visible; 2 = swollen foot (dorsally visible) [27]. For toe damage: 0 = no damage; 1 = missing nails or toe deformities; 2 = one or more fingers missing.

#### 2.3.2. Egg Distribution and Quality

To estimate whether different levels of freedom of movement affected nest use and nest preference, a sectional egg count was carried out on the conveyor belt. To account for between-days variability, the differential egg count was carried out on two consecutive days at each timepoint, and the average data were used. In short, the conveyor belt of each tier and side (right vs. left) of each aviary was divided into 11 sections, and the number of eggs in each section was counted. Each tier and side of the aviary had five nest (N) and six outside nest (ON) sections that were identified based on the position (in front of a nest = N and not in front of a nest = ON) on the days of the egg count. The sections of the conveyor belt in front of a nest were named “N” and numbered progressively starting from the main entrance door of the building and according to the direction of the ventilation within the building (sections from N1 to N5, see also Figure S1 in the Supplementary Materials). Sections not in front of a nest were named ON and numbered according to the same criterion (from ON1 to ON6). Eggs laid on the litter were not considered because they were a meagre number (less than 10 per week, according to the farm personnel).

During the first of each two consecutive sectional count days, a sample of 180 eggs per aviary was collected from the conveyor belt and transferred outside the barn for the subsequent egg quality assessment. Eggs were randomly sampled from the three tiers of each aviary (N = 60 per tier) and along the entire length of the aviary, picking up eggs proportionally from both the N and the ON sections.

Egg defects were visually observed by the same group of trained assessors, who collectively observed one egg at a time to provide a consistent evaluation throughout the trial. The following egg defects were noted down: broken (gross cracks, with exposure of the internal content); cracked (hairline crack, without exposure of the internal content); dirty (presence of faeces or debris from the egg conveyor belt); sandpaper or rough shells (presence of calcium deposits on the shell); porosity (thin shell or parts of it, with a translucent appearance); blood stains on the shell; and other defects (including misshapen eggs, extreme size differences such as very large or tiny eggs, shell-less eggs, scratched shells, etc.). The overall number of eggs not suitable to be sold in their shells (broken, dirty or with blood stains) was also recorded. Eggs having more than one defect were counted in both categories. Therefore, the total percentage sometimes exceeds 100%.

#### 2.3.3. Behavioural Observations

Two IP cameras suitable for day and night vision (MiniDome model, Zhejiang Dahua Technology Co. Ltd., Hangzhou, China) were installed inside each aviary. To guarantee a good vision of the two sides and the three floors of the aviary, cameras were installed in the central corridor of the aviaries, on the net separating two adjacent aviaries. One of the cameras was placed at about 2 m height from the floor, the other at about 0.50 m height. Cameras were covered with a plastic protective shield to avoid pecking. They were cleaned immediately before beginning each of the video recording sessions to eliminate visual obstacles due to dirt.

Animals were video recorded over 24 h on the four occasions mentioned above, and videos were stored on a secure server. At the end of the trial, video recordings were analysed using a video-surveillance software (Milestone Xprotect Expert 2019 R2, Milestone Systems, Brøndby, Denmark). Similar to what Stratmann et al. [28] described, behavioural observations were carried out by analysing the videos for a total duration of 15 min per day, distributed as follows:

- Morning: from 07:05 to 07:10 a.m. (i.e., the minutes after the full brightness of illumination inside the aviary was achieved);
- Mid-day: from 2:00 to 2:05 p.m. (i.e., after feed distribution, when animals are calmer, and expected to rest or dustbathe);
- Evening: from 6:55 to 7:00 p.m. (i.e., the minutes when lights in the aviary started to be switched off, starting from the lower tier).

Videos were analysed in slow motion by three trained observers, who carried out the behavioural analysis collectively to avoid inter-observer variability, and due to the high number of animals on video to ensure that all the relevant behaviours were noted down. The following behaviours were analysed (the definitions from Stratmann et al. [28] were used to identify behaviours):

- Flights and flight attempts: this observation included the departure and the arrival tier (to distinguish flights as vertical—across different tiers—or horizontal—with departure and arrival at the same level of the aviary—);
- Walking (along the aviary and on the ramps);
- Collisions against aviary fixtures, perches, other hens or the floor (falls o incorrect landings).

#### 2.3.4. Statistical Analysis

Statistical analysis was carried out using the software Statistica v.12 (StatSoft Inc., 2014).

The variables were analysed using analysis of variance, with the experimental group and floor of the building as the main effects. The individual (hen or egg) was used as the experimental unit.

Dunnett test was used for pairwise comparisons between the control group and each of the groups with structural modifications (LM, VM, FM).

A Chi-square test was used to compare class distribution for BCS, KBD and plumage damage. For plumage damage, classes 1–2 (mild damage) and 3–4 (severe damage) were aggregated.

Statistical significance was set at *p* < 0.05.

## 3. Results

Animal weight and mortality (data not shown) recorded by the farm personnel were in perfect agreement with the recommendations for the specific hybrid [29]. Mortality was, on average, 6.2% over the entire cycle, with no significant difference among the experimental groups.

### 3.1. Welfare Indicators

Overall, the average score and the class distributions calculated across the trial for the welfare indicators showed no significant difference across the experimental groups (*p* > 0.05, data not shown). However, for some parameters, an effect of the timepoint was observed. In the case of plumage damage, a worsening of the conditions (i.e., higher scores) was observed in all body areas as time progressed.

The results obtained at the last timepoint (W78) for BCS, KBD and Plumage damage are shown in Table 1 (average scores) and in Table 2 (class distribution).

At the end of the trial, KBD did not show statistical differences among groups. At the same time BCS was significantly higher (i.e., lower presence of lean animals) in the C group compared to all the other groups. For what concerns plumage damage, significant differences among the four experimental groups were detected for all the body areas, with the only exception being wings. The pairwise comparisons (Dunnett test) show that the effects of the experimental group on plumage differed depending on the body region. Feathers in the neck region were in better conditions in C than in LM and VM, but similar to FM; feathers in the breast area were in worse conditions in C compared to the FM group; feathers in the back region were in better conditions in FM and worse in VM group compared to C and the same was true also for the average score of the whole body; lastly, feathers in the tail region were significantly better both in the LM and in the FM group compared to control.

To facilitate the understanding of the class distributions (Table 2), the statistically different variables (BCS, neck, back, tail) are also shown graphically in the Supplemental Materials (Figure S6). The chi-square statistics (Table 2) confirm most of the differences already observed in terms of average scores. In particular, the analysis of the class distributions confirms the direction of the statistical difference for BCS (class distribution showing fewer lean animals in C than in the three modified cages) and for plumage in the back region (worse scores were observed in VM and better scores in FM compared to C). For plumage conditions in the neck and tail region, only some of the differences are confirmed (in the neck region, worse scores were observed for LM and VM groups; in the tail region, worse scores were observed in the VM group and better scores were observed in the FM group compared to C), and two additional differences appeared (better feather scores observed in FM in the neck region and in LM in the tail region compared to C). All differences observed by this analysis were highly significant (*p* < 0.001).

For what concerns foot conditions (data not shown), the number of lesions observed was extremely low, and no statistically significant difference was found (89 bumblefoot and 22 toe lesions over the 2240 animals assessed during the whole trial, corresponding to a 4% and 1% prevalence, respectively).

### 3.2. Egg Distribution and Quality

Regardless of the experimental group, hens showed a strong preference for laying in the higher levels of the aviary compared to the lower ones (across the entire trial, 46.9% of the eggs were laid in the top tier, 37.5% in the middle one and 15.6% in the lower one), with a significant difference (*p* < 0.01) among the three levels (data not shown). However, the difference in the proportion of eggs laid in each tier did not affect the percentage of eggs laid inside/outside the nests (87.3%/12.7% in the top tier, 93.3%/6.7% in the middle tier and 89.4%/10.6% in the lower tier, *p* > 0.05, data not shown).

Results show no statistical difference among groups in the egg distribution pattern, or for the distribution across the tiers nor in the proportion of mislaid eggs (i.e., eggs laid outside the nest) (Table 3). However, an interesting, although merely numerical, difference can be observed in the VM group: with the ramps available, these animals laid more eggs in the higher tier of the aviary. However, this trend was not observed in FM. It can also be observed that in VM the highest concentration of eggs on a single floor is combined with the highest proportion of eggs laid outside the nests.

The graphs on the egg distribution (Figure S7, available in the Supplemental Materials) show great variability across the different nests, without highlighting any clear pattern.

With respect to egg defects, most of them were found to increase significantly as the trial progressed, regardless of the experimental group. This trend was observed for sandpaper, dirty, cracked, broken-pecked and porous eggs (*p* < 0.001, data not shown). Moreover, the tier of the aviary affected egg quality, with eggs laid on the top floor showing more defects, especially being dirtier (*p* < 0.001), more broken-pecked (*p* < 0.05) and having an overall higher percentage of eggs not suitable for being sold (dirty + broken, *p* < 0.001, data not shown) which was likely related to the higher proportion of eggs laid in this floor. By contrast, Table 4 shows that the experimental group did not affect egg quality.

### 3.3. Behavioural Observations

Regardless of the experimental group, hens carried out significantly more displacements in the morning (80.0 displacements observed in 5 min on average) compared to the afternoon and evening observation sessions (47.2 and 45.5 displacements, *p* < 0.001, data not shown). Of the displacements, horizontal flights were more frequent in the mornings (32.2% of the displacements) and vertical flights in the evenings (85.4%) compared to the other moments of the day (*p* < 0.001, data not shown).

Concerning the behavioural observations, while the number of total displacements observable on camera did not significantly differ across groups, the kind of displacements significantly differed between groups not having ramps (C and LM) and groups having them (VM and FM) (Table 5), with groups having ramps carrying out a significantly higher proportion of walks and a significantly lower proportion of flights compared to groups without ramps (*p* < 0.05). The structural modifications of the aviary did not alter the proportion of vertical vs. horizontal flights or the prevalence of falls, collisions, or incorrect landings, which resulted in being quite low across the trial. With respect to the use of ramps, VM hens used them significantly more than FM hens when walking along the aviary (*p* < 0.01).

## 4. Discussion

### 4.1. Welfare Indicators

While the welfare indicators calculated during the entire trial did not show significant variations among groups, some differences were observed at the last timepoint. This fact indicates that the chronic effect of the structural modifications of the aviaries may become more evident at the end of the productive life of the animals, when it is summed up with the high metabolic demand of an extended production cycle [30]. As time progressed, a worsening in feather conditions had previously been observed also by Bilcik et al. [31] (although their study stopped at 33 weeks of age), and very recently also by Schwarzer et al. [32] (until approximately 70 weeks of age). Similar observations were also made for KBD [33].

At the end of the trial, the control group showed significantly higher BCS scores compared to animals having more freedom of movement. This effect could at least partially be due to the increased energy expenses of animals being freer to roam inside the aviary (regardless of the spatial direction), although the number of displacements visible on camera did not significantly increase. However, this may warrant the need to monitor more closely the nutritional needs of hens in aviaries with increased freedom of movement. On the other hand, it is also possible that animals may have overcrowded some areas of the aviary, increasing the competition to access resources, including feed, in those areas and resulting in more lean animals [4]. In this sense, at least some partitions may have a positive effect in increasing the uniformity of animal distribution. Similar observations can also be made for feather damage. Overall, animals with more freedom to move vertically showed worse plumage conditions (especially in the neck, back and tail region), while animals with complete freedom of movement showed an improvement in feather conditions (especially in the breast, back and tail region) compared to the control group. The back, breast and neck regions are largely acknowledged in the literature as the areas in which feather pecking lesions are observed more frequently [31,32,34,35,36]. This increase in feather pecking behaviour in VM and reduction in FM compared to the control group, however, highlights an inconsistent effect of ramps on animal welfare, seeming to indicate that pecking is more present when vertical movement is allowed but lateral movement is limited (VM group), therefore in this group the (relatively) higher density and competition, although only in localized areas delimitated by partitions and likely at the higher end of the ramps, may have triggered feather pecking behaviour [37,38].

With respect to KBD, the high prevalence of lesions at the end of the trial is compatible with the progressive increase in keel lesions observed by Casey-Trott et al. [33]. In contrast with other recent studies [20,28], we failed to find a mitigating effect of the installation of ramps on the risk of keel bone damage. It should, however, be highlighted that the aviaries used in the mentioned studies (non-overlapping tiers, with resources distributed only on specific tiers) were significantly different compared to the combi system tested in the present study. This lack of a positive effect of the ramps could also be because the distance between the two sides of the aviary was approximately 0.90 m, which, according to some Authors [26,39], facilitates hens’ flight. On the other hand, we did not observe any worsening in KBD prevalence, meaning that the ramp design was adequate and the ramps themselves did not determine an increased risk of collisions/failed landings. Similarly, ramps did not increase foot lesions, contrarily to the rise in hyperkeratosis observed by Weitzenbürger et al. [40].

### 4.2. Egg Distribution and Quality

Regardless of the experimental group and the time point, hens showed a high preference for laying in the higher tier; this agrees with recent observations on white hens from Purdum et al. [41], who hypothesized that it might be a result of a more remarkable ability to reach the top tier and greater motivation to lay at higher levels compared to brown lines.

Overall, mislaid (outside nest) eggs were a low percentage (similar to previous observations by Appleby et al. [42]) and were not affected by the structural modifications. Despite the absence of statistical differences in egg distribution patterns, in the VM group the highest proportion of eggs was laid in the elevated tier and was associated with the highest percentages of eggs laid outside the nests and egg defects. Similar results were found very recently also by van den Oever et al. [43], who observed that a more uneven nest distribution was positively correlated with a higher percentage of mislaid (floor) eggs. This fact may depend on the mentioned overcrowding of some areas of the aviary, which may have forced more hens to lay outside the nest area, suggesting insufficient space for oviposition in nests in the preferred areas [44] or the occurrence of gregarious nesting [43]. Although not significant, this finding seems to indicate that when both ramps and partitions are present, animals use the ramps to reach the elevated floors of the aviary but might then not be able to negotiate a suitable place for oviposition.

Regardless of the experimental group, the significantly higher incidence of egg defects (especially dirty and broken eggs) observed in the elevated tier is likely related to the relative overcrowding and the higher proportion of eggs laid in this tier. That may have increased the possibility that eggs could become dirty due to contact with other hens’ bodies or feet, that they may be pecked by other hens [42,45], or cracked when colliding with other eggs if several eggs are laid in the same area. However, van den Oever et al. [43] also failed to find a correlation between the percentage of broken eggs and the gregariousness of nesting behaviour, contrary to the Authors’ expectation.

Egg distribution across the nests of the aviary did not highlight any clear pattern; since it was characterized by extreme variability, it can be argued that the nest preference might vary depending on factors that were not controlled in the present study (for example, the microclimate inside the single nest or social facilitation [44]).

From a general standpoint, egg deposition seemed to show more uniformity across the tier when partitions were present (C and VM groups), possibly indicating that partitions allowed animals to create subgroups and distribute more evenly across the entire length of the aviary. In contrast, when they were not present (LM and FM groups), hens seemed to prefer the nests close to one of the partitions separating two subsequent aviaries (namely N1 and N5). A general preference for corner and end-of-the-line nest boxes was previously suggested by Riber [46], possibly because they are perceived as more isolated and enclosed or that they may also be considered different and easier to locate than centrally located nest boxes.

### 4.3. Behavioural Observations

Regardless of the experimental group, the fact that hens carried out more horizontal displacements in the morning and more vertical displacements in the evenings is compatible with their natural behaviour (looking for a nest at dawn and perching at sunset) [28].

The absence of statistical differences among groups in the number of total displacements observed on camera seems to indicate how the structural modifications did not affect the displacements carried out by the hens. However, in groups where the partitions were removed (VM and FM), animals could carry out displacements (walking) also inside the aviary tiers, and these walks could not be recorded on camera. Therefore, the behavioural observations in these groups provide only a partial representation of the displacements carried out by the animals. In addition, due to the commercial conditions in which the trial was carried out, it was possible to observe only a limited part of the aviary and not its entire length, and the animal density sometimes made it challenging to discriminate displacements happening far away from the camera. For these reasons, behavioural observations should be interpreted with caution. Similar difficulties in interpreting hens’ movement were also found by Rufener et al. [47], who used infrared receivers attached to the legs of focal hens to record their movements.

Despite these limitations, we observed that when ramps were available, hens used them to walk and carried out significantly fewer flights than groups without ramps (flights remained, however, the most common displacement method). On the other hand, in the FM group hens used the ramps less than in the VM group; however, it cannot be excluded that without partitions, animals may have increased their displacements (walking) along the aviary (intratier). From a general standpoint, Norman et al. [23], observed that flocks reared with ramps also in the pullet farm showed increased use of the elevated structures and lower hesitancy in ramps use during the laying period, indicating specific learning during the early life experience that translated and persisted in later life. This could be a valuable strategy to promote ramp use and improve animal health also later in life. As previously mentioned, very recent findings from Stratmann et al. [21] also described a high voluntary use of ramps and a better distribution of chicks across the aviary tiers.

Lastly, as confirmed by the absence of differences in KBD, the prevalence of falls, collisions or incorrect landings was not affected by the structural modifications. This result contrasts with what was reported by Stratmann et al. [28], who observed a reduction in all these issues when ramps were installed. Interestingly, Rufener et al. observed that the total number of transitions was not linked to the severity of KBD [47].

## 5. Conclusions

Our study tested the application of structural modifications (increased lateral and/or vertical freedom of movement) in a commercial setting, although with some limitations in the possibility of accurately carrying out behavioural observations. Overall, an inconsistent effect of the structural modifications was observed, with hens showing worse body conditions in all the aviaries with increased freedom of movement, while plumage conditions were improved in the freedom of movement group but worsened in the vertical movement group. No significant effect on egg deposition pattern, egg quality or keel bone damage was observed, even though animals seemed to use the ramps when available. Overall, apart from some overcrowding and feather pecking concerns in the vertical movement group (where the combination between ramps and the presence of partitions may have increased overcrowding in specific areas), the other two modifications (lateral movement and freedom of movement) seemed to facilitate the possibility for animals to move across different areas of the aviary and carry out their species-specific behaviour. However, it would be extremely interesting to monitor more closely how animals move three-dimensionally in the aviary by following focal animals or evaluating the displacements carried out inside the aviary tiers (intratier).

## Figures and Tables

**Table 1:** Average scores of the welfare parameters assessed at the end of the trial (78 weeks of age) (C = Control, LM = Longitudinal Movement, VM = Vertical Movement, FM = Freedom of Movement, BCS = Body Condition Score, KBD = Keel Bone Damage). The whole body score was calculated as the average of all the body areas assessed. *p*-value: indicates the statistical significance of the differences among the 4 experimental groups, considered altogether. Dunnet test *p*-values: they indicate the significance of differences between the control group and each of the groups with structural modifications, taken individually (C vs. LM, C vs. VM and C vs. FM).

			Plumage Damage						
**Group**	**BCS**	**KBD**	**Neck**	**Breast**	**Belly**	**Back**	**Wings**	**Tail**	**Whole Body**
**C**	1.06	1.28	0.78	2.51	1.99	1.39	0.59	1.53	1.46
**LM**	0.86	1.20	1.19	2.37	2.16	1.50	0.61	1.11	1.49
**VM**	0.94	1.34	1.04	2.44	2.24	1.67	0.71	1.74	1.64
**FM**	0.85	1.14	0.75	2.15	1.92	1.11	0.56	1.08	1.26
***p*** **-value**	0.001	n.s.	0.001	0.01	0.05	0.001	n.s.	0.001	0.001
**Dunnet test *p*-values**									
**C vs. LM**	0.001	n.s.	0.001	n.s.	n.s.	n.s.	n.s.	0.001	n.s.
**C vs. VM**	0.05	n.s.	0.05	n.s.	n.s.	0.05	n.s.	n.s.	0.05
**C vs. FM**	0.001	n.s.	n.s.	0.01	n.s.	0.05	n.s.	0.001	0.05

**Table 2:** Class distribution (expressed as a percentage) of the welfare parameters assessed at the end of the trial (78 weeks of age) (C = Control, LM = Longitudinal Movement, VM = Vertical Movement, FM = Freedom of Movement). For plumage damage, classes 1–2 (mild damage) and 3–4 (severe damage) were aggregated. The symbol § close to the class distribution percentages indicates significant differences with the control group.

	Group	C	LM		VM		FM		Significance Level (*p*<)
	Class								
**Body Condition Score**	0	7	15	§	10.7	§	15	§	0.001
	1	80	84.3		85		85		
	2	13	0.7		4.3		0		
**Keel Bone Damage**	0	19.3	22.9		15.7		28.6		n.s.
	1	33.6	35		34.3		28.6		
	2	47.1	42.1		50		42.8		
**Plumage damage**									
**Neck**	0	36.4	24.3	§	37.9	§	50	§	0.001
	1–2	62.1	63.6		51.4		44.3		
	3–4	1.5	12.1		10.7		5.7		
**Breast**	0	6.4	3.6		5.7		7.9		n.s.
	1–2	32.9	44.2		35		47.1		
	3–4	60.7	52.2		59.3		45		
**Belly**	0	17.1	11.4		10.7		16.4		n.s.
	1–2	37.1	35.7		30.7		42.,8		
	3–4	45.7	52.8		58.5		40.7		
**Back**	0	25.7	40	§	15	§	44.3	§	0.001
	1–2	55.0	20		57.8		37.2		
	3–4	19.3	40		27.1		18.6		
**Wings**	0	51,4	50.7		45.7		53.6		n.s.
	1–2	48.6	49.3		54.3		46.4		
	3–4	0	0		0		0		
**Tail**	0	10.7	32.9	§	13.6	§	27.1	§	0.001
	1–2	77.9	60		65.7		64.3		
	3–4	11.4	7.1		20.7		8.6		

**Table 3:** Egg distribution (expressed as a percentage of counted eggs) in terms of nest use (N = nest or ON = outside nest), across the tiers on the aviary (top, middle and lower tier) and across the experimental groups (C = Control, LM = Longitudinal Movement, VM = Vertical Movement, FM= Freedom of Movement).

	Per Group				Sign. Level (*p*<)
	C	LM	VM	FM	
**Tier**					
**Top**	47.5	44.1	50.5	45.6	n.s.
**Middle**	35.5	37.7	37.8	39.1	
**Lower**	17.0	18.2	11.7	15.3	
**Eggs laid in N** **(eggs laid ON)**	91.5 (8.5)	92.2 (7.8)	87.4 (12.6)	89.0 (11.0)	n.s.

**Table 4:** Egg defects (expressed as a percentage) across the entire trial (C = Control, LM = Longitudinal Movement, VM = Vertical Movement, FM = Freedom of Movement). Significance is assessed across groups, timepoints and tiers. At each timepoint, two aviaries per group were assessed (180 eggs sampled per aviary, of which 60 from each tier).

	Group				Sign. Level (*p*<)
%	C	LM	VM	FM	
**No defects**	58.7	53.2	59.4	53.2	n.s.
**Sandpaper or rough shell**	18.7	19.3	17.7	18.2	n.s.
**Blood stains**	0.7	0.8	0.8	1.3	n.s.
**Dirty**	25.2	27.1	28.1	21.9	n.s.
**Cracked**	6.7	8.1	5.7	5.9	n.s.
**Broken-pecked**	3.3	4.0	3.3	4.1	n.s.
**Other defects**	0.1	0.6	0.7	0.5	n.s.
**Thin/porous shell**	4.9	7.1	4.4	5.5	n.s.
**Not for sale (dirty + broken)**	33.4	34.8	33.9	27.4	n.s.

**Table 5:** Behavioural observations on the movements across the trial (2 replications per group) and at different times of the day. C = Control, LM = Longitudinal Movement, VM = Vertical Movement, FM = Freedom of Movement. (A, B: different superscripts on the same row indicate a statistically significant difference between groups).

	Group				Sign. Level (*p*<)
	C	LM	VM	FM	
**Displacements in 5 min. (nr)**	58.2	49.8	58.7	52.9	n.s.
**Flights (%)**	99.4 ^A^	99.6 ^A^	90.7 ^B^	93.9 ^B^	0.05
**Walks (%)**	0.64 ^B^	0.40 ^B^	9.3 ^A^	6.1 ^A^	0.05
**Horizontal flights (%)**	25.7	25.0	23.7	20.7	n.s
**Vertical flights (%)**	74.3	75.0	76.3	79.3	n.s
**Falls, collisions or incorrect landings (%)**	0.58	0.41	1.21	0.71	n.s
**Ramp use (% of walks)**	/	/	90.4 ^A^	68.9 ^B^	0.01

## Data Availability

Restrictions apply to the availability of these data. Data was obtained under a partially funded research project and are available from the authors with the permission of Eurovo Group.

## Supplementary Materials

The following supporting information can be downloaded at: https://www.mdpi.com/article/10.3390/ani12182307/s1.
