# Supplementary material for: Influence of Increased Freedom of Movement on Welfare and Egg Laying Pattern of Hens Kept in Aviaries
Source: Animals (Basel). 2022 Sep 6;12(18):2307. doi: 10.3390/ani12182307 (PMC9495151; doi:10.3390/ani12182307)

Supplemental Material

Figure S1: Schematic representation of the aviary system used in the experimental trial: a) view from above; b) Transversal section

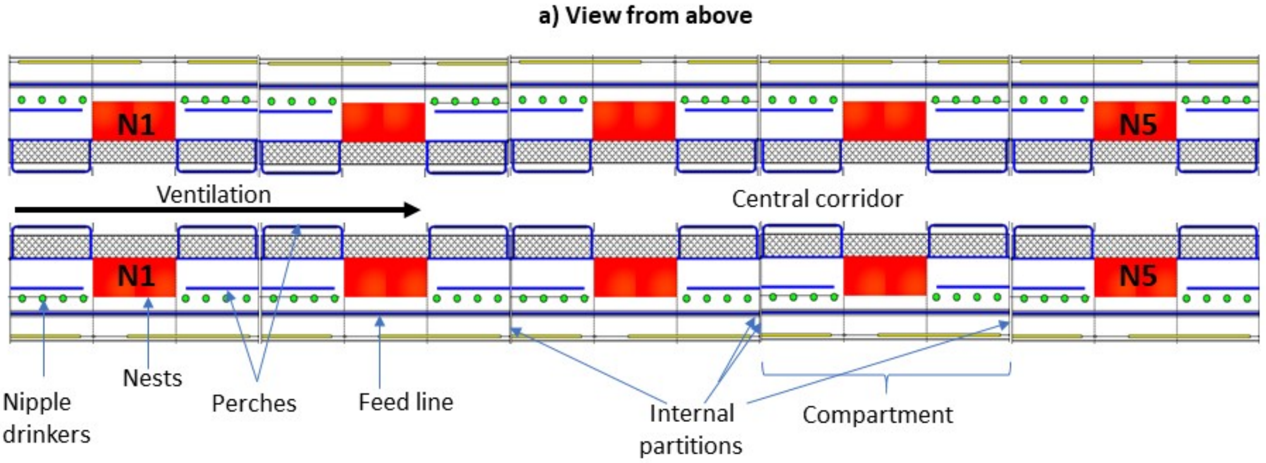

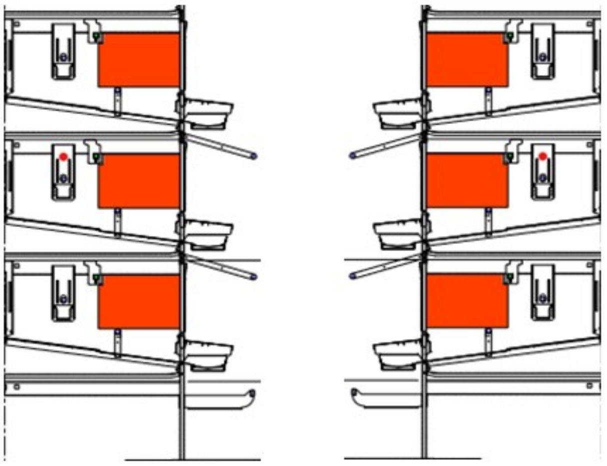

Figure S2: Aviaries used in the Control (C) group: Presence of the transversal partitions (4 per tier and side of the aviary) dividing each tier and side into 5 compartments. The red arrow shows on of the partitions.

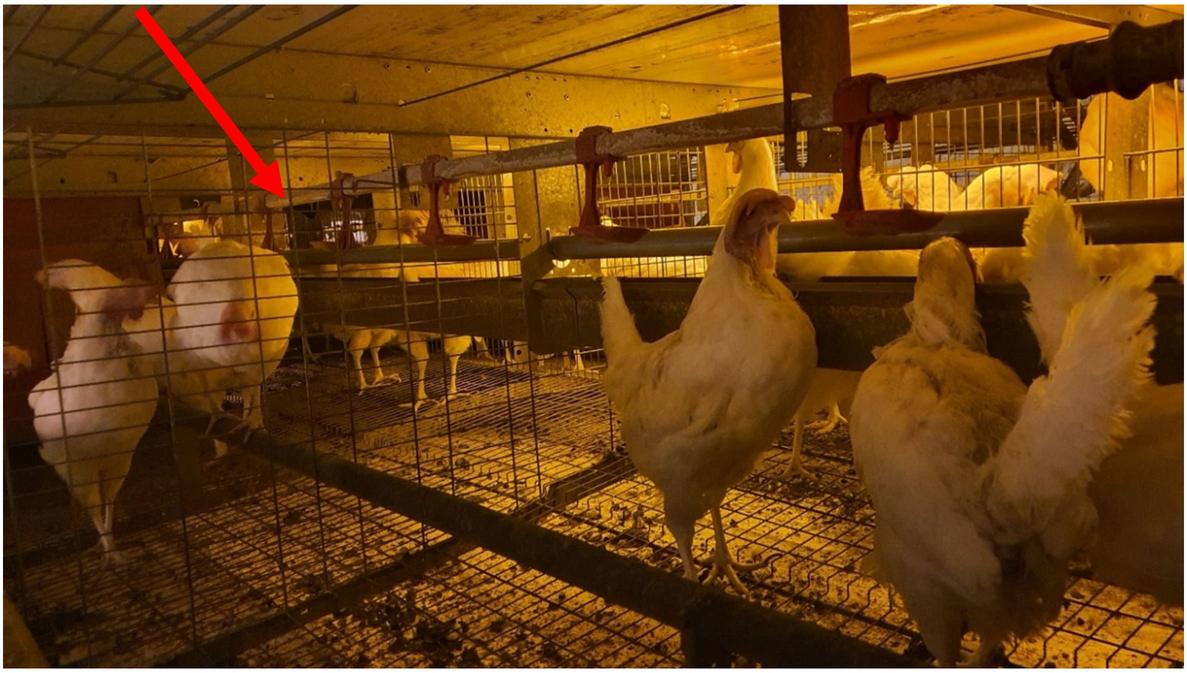

Figure S3: Aviaries used in the Lateral Movement (LM) group. The red arrow shows the point where one of the transversal partition was removed.

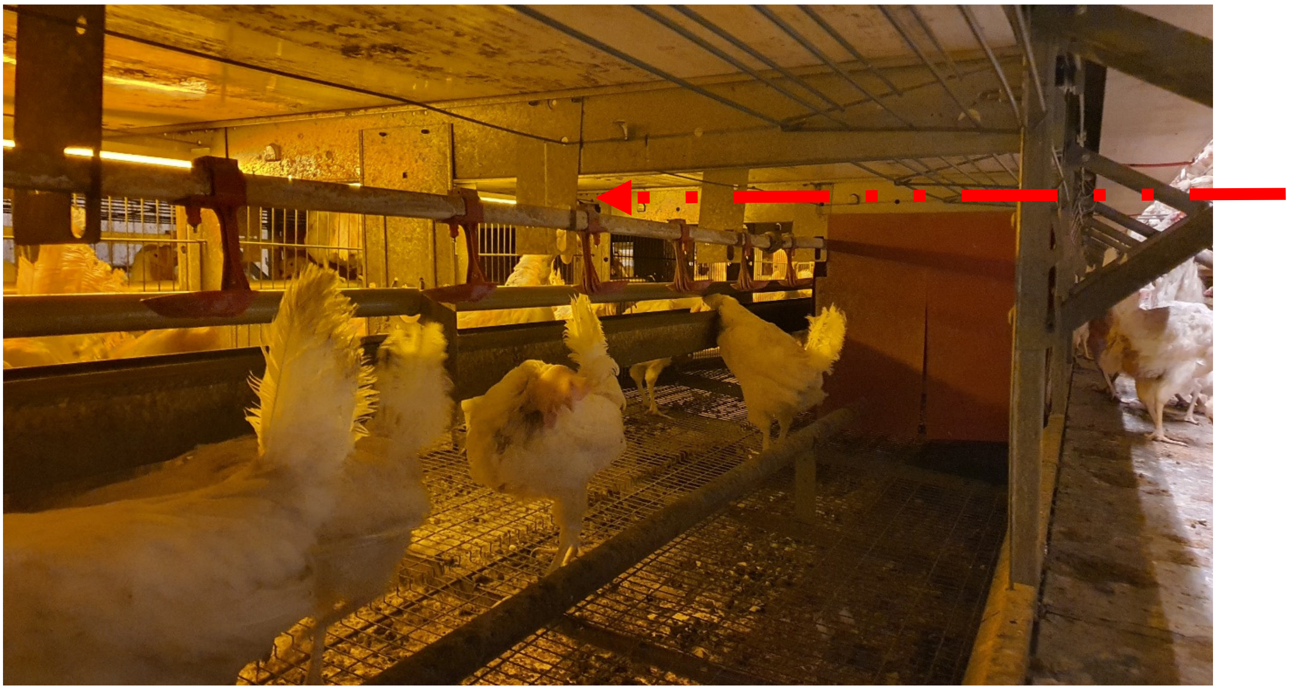

Figure S4: Aviaries used in the Vertical Movement (VM) group: detail of one of the metal ramps that were installed between the different tiers of the aviary.

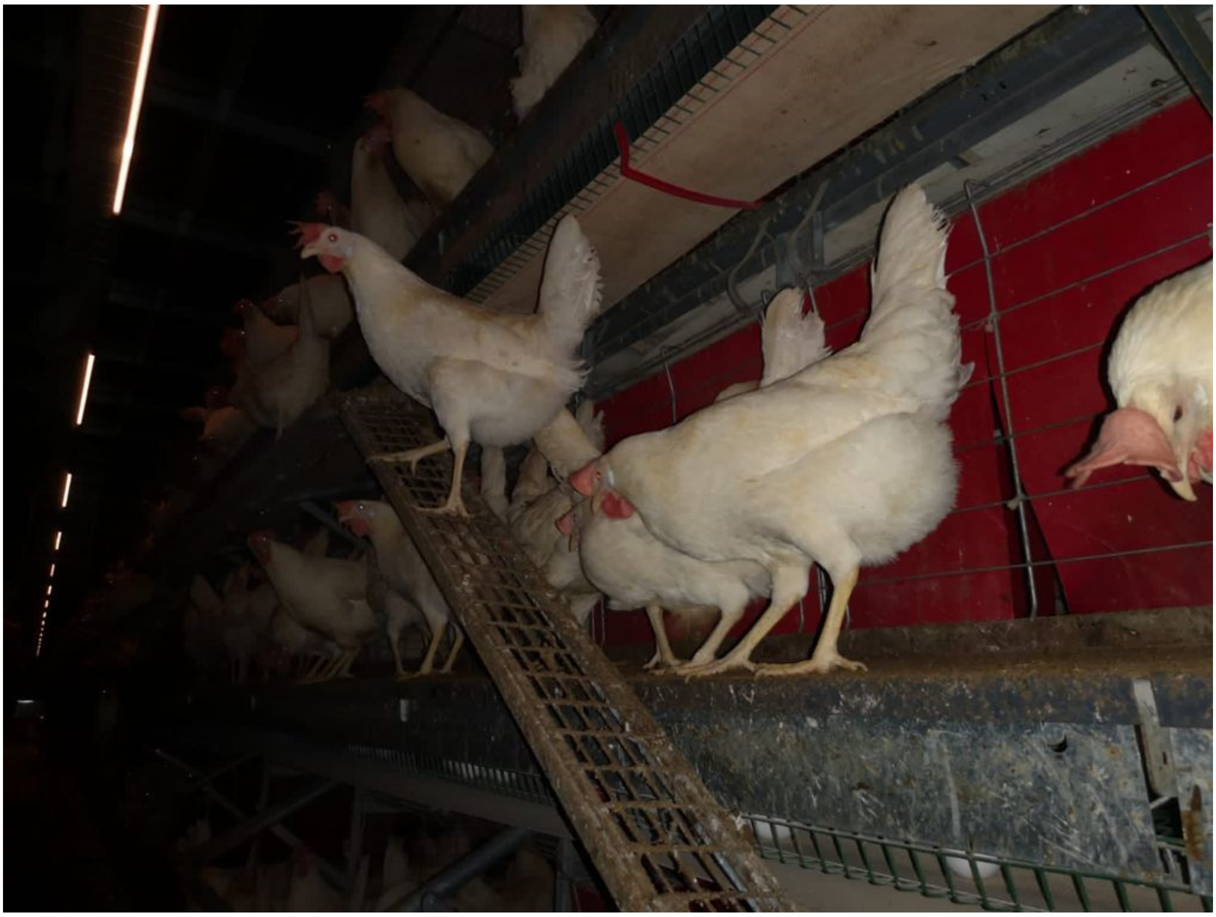

Figure S5: Overall view of the empty aviary with the ramps installed

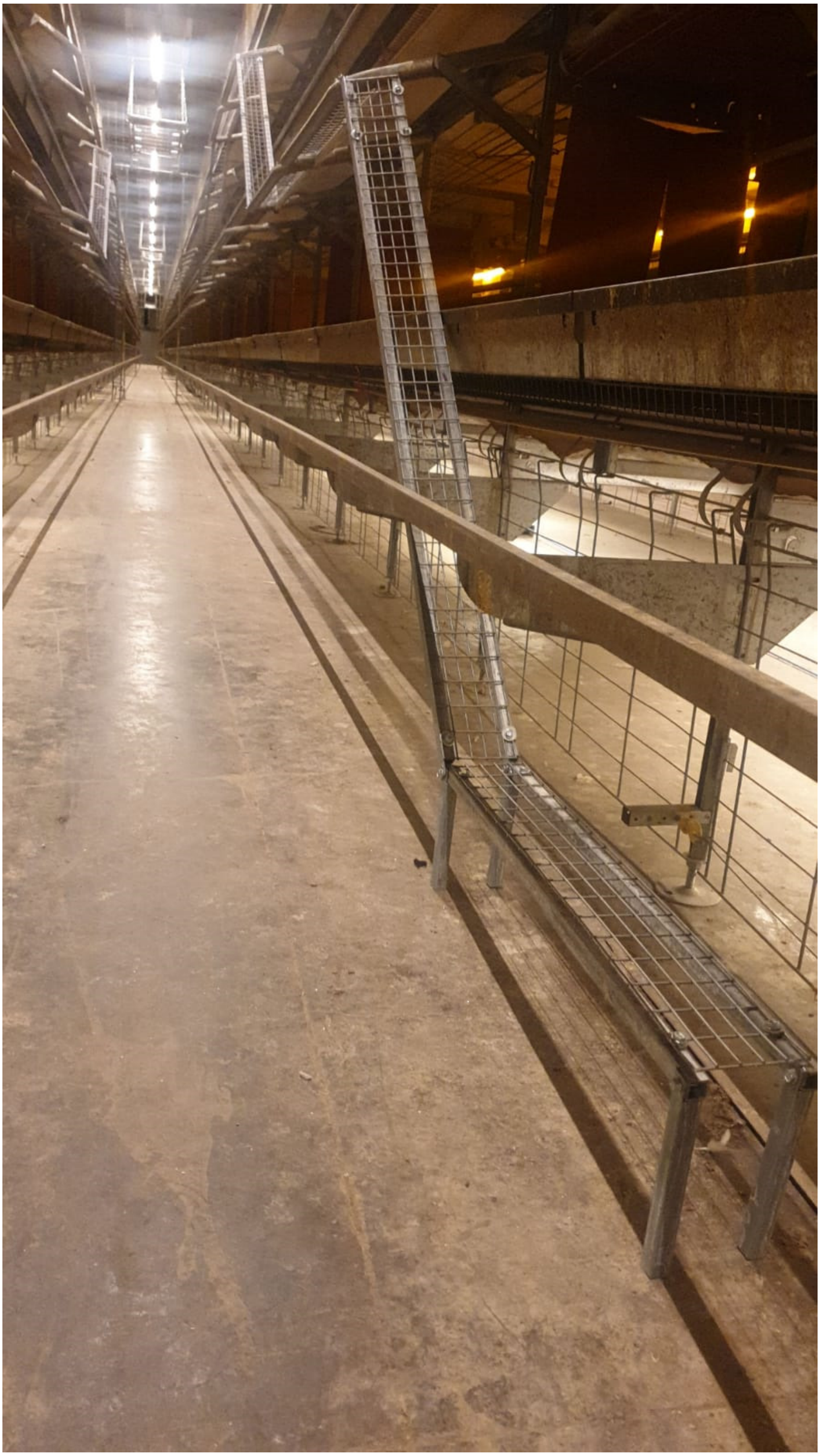

Figure S6: Graphical representation of the class distribution (%) for the statistically significant parameters highlighted in Table 2

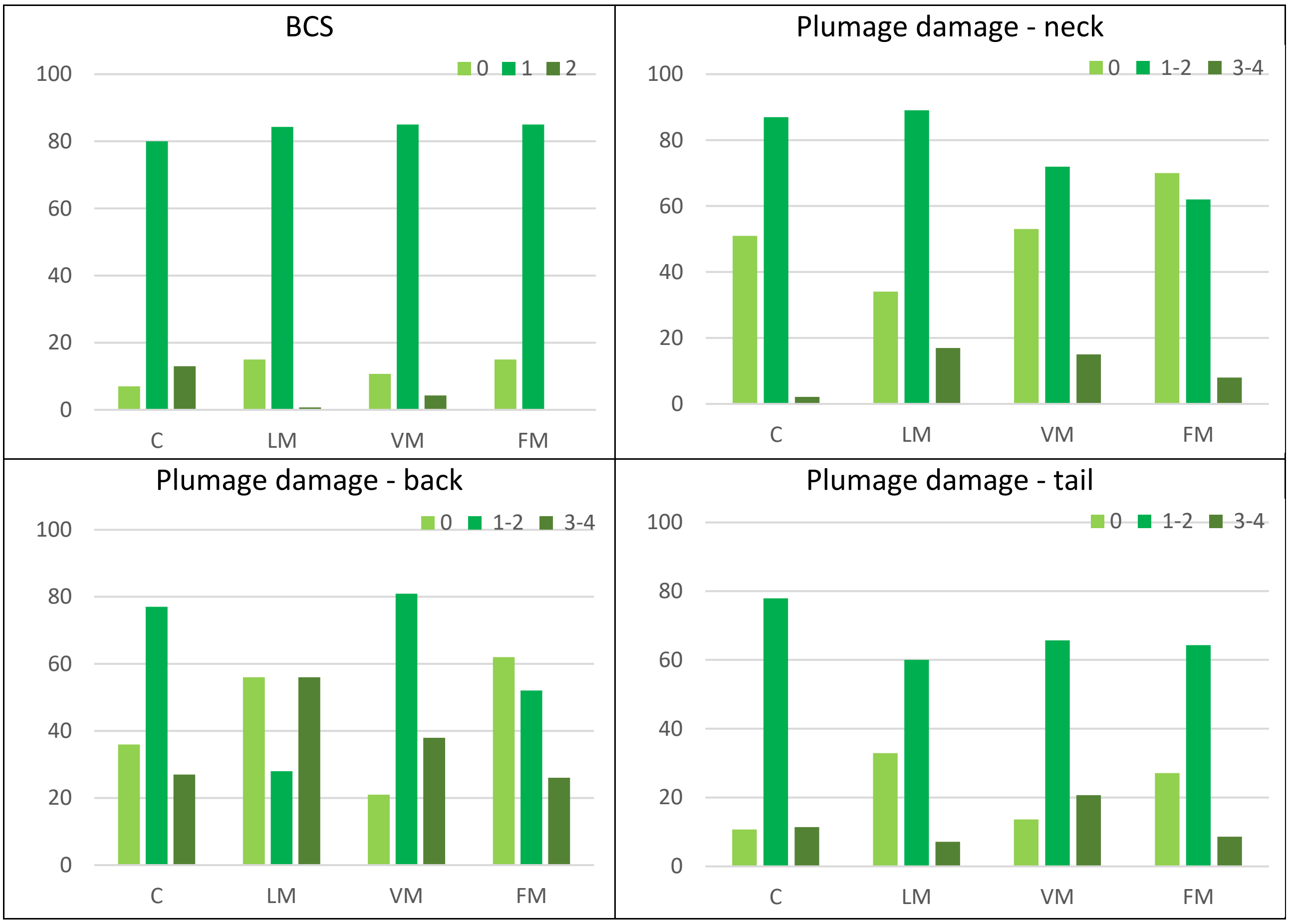

Figure S7: Egg distribution (expressed as a percentage) in the experimental groups across the nests over the entire trial (percentage ± standard deviation). C=Control, LM=Longitudinal Movement, VM=Vertical Movement, FM= Freedom of Movement, top, middle and lower tier of the aviary. Nests were numbered from 1 to 5 starting from the main entrance of the building and according to the direction of the ventilation (See figure S1).

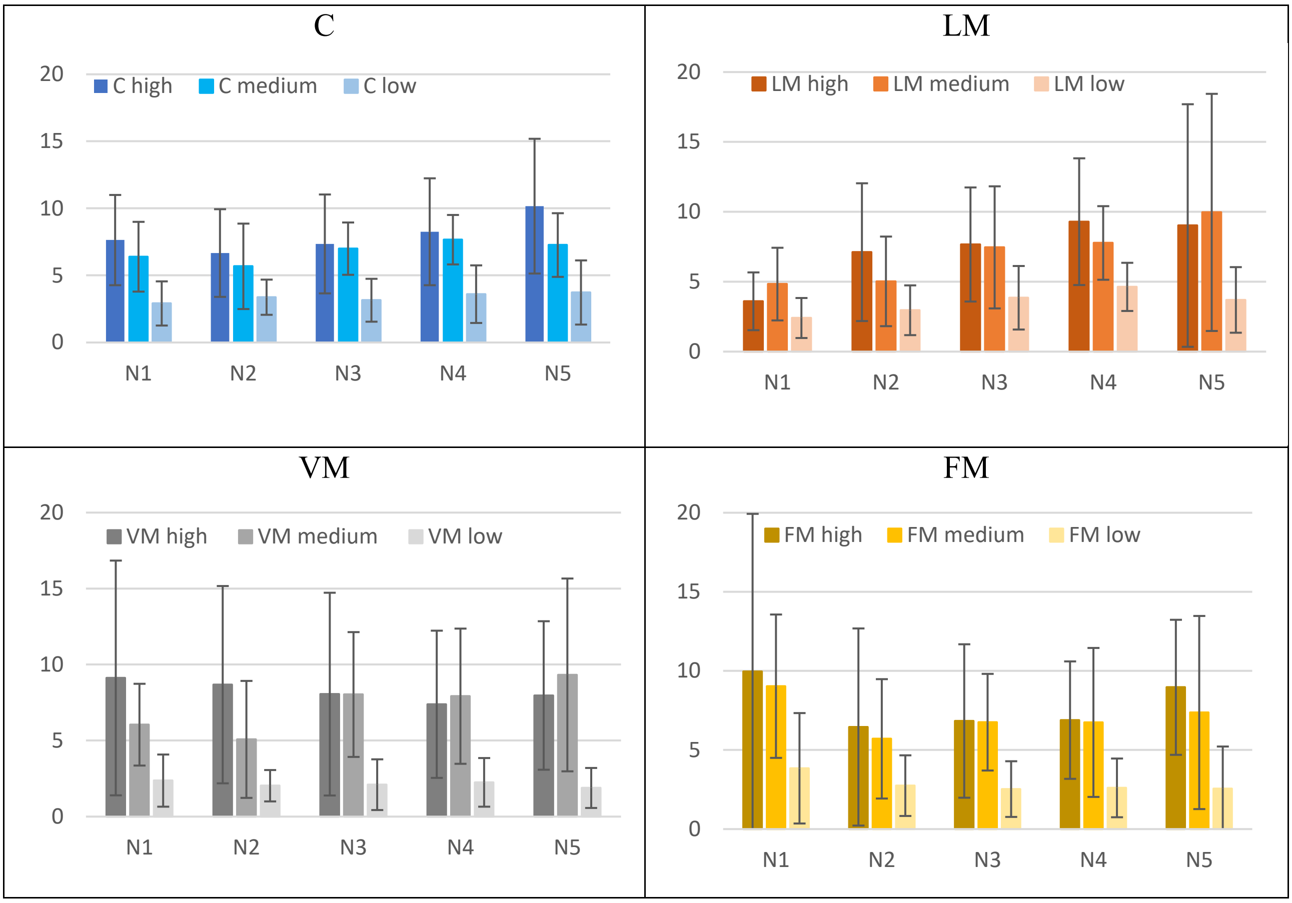

Supplement: Supplementary file 1 [file animals-12-02307-s001.zip › animals-1876715-supplementary.pdf]
